# Prospective, Multicentre Feasibility Study of Remote Colon Capsule Endoscopy Using the OMOM CC100 System

**DOI:** 10.3390/diagnostics16010020

**Published:** 2025-12-20

**Authors:** Alexandra Agache, Ervin Toth, Niels Qvist, Miguel Mascarenhas, Wojciech Marlicz, Benedicte Schelde-Olesen, Miguel Mascarenhas-Saraiva, Maria Marlicz, Gabriele Wurm Johansson, Artur Nemeth, Anastasios Koulaouzidis

**Affiliations:** 1 Department of Clinical Research, University of Southern Denmark (SDU), 5000 Odense C, Denmark; alexandra.agache@rsyd.dk (A.A.);; 2 Department of Surgery, Odense University Hospital (OUH), 5000 Odense C, Denmark; niels.qvist@rsyd.dk; 3 Department of General Surgery, University of Medicine and Pharmacy ‘Carol Davila’, 050474 Bucharest, Romania; 4 Department of Gastroenterology, Skåne University Hospital, 205 02 Malmö, Sweden; ervin.toth@med.lu.se (E.T.); gabriele.wurmjohansson@skane.se (G.W.J.); artur.nemeth@skane.se (A.N.); 5 Department of Clinical Sciences Malmö, Lund University, 22100 Malmö, Sweden; 6 Gastroenterology, Centro Hospitalar São João, 4200-319 Porto, Portugal; miguelmascarenhassaraiva@gmail.com; 7 WGO Gastroenterology and Hepatology Training Center, 4200-437 Porto, Portugal; 8 The Centre for Digestive Diseases, Endoklinika sp. z o.o., Kurza Stopka 3,4, 70-535 Szczecin, Poland; wojciech.marlicz@hotmail.com (W.M.); mariamarlicz@gmail.com (M.M.); 9 Department of Gastroenterology, Pomeranian Medical University, Unii Lubelskiej 1, 70-204 Szczecin, Poland; 10 ManopH, Digestive Endoscopy & Motility Lab., 4000-432 Porto, Portugal; miguelms.manoph@gmail.com

**Keywords:** capsule endoscopy, colonoscopy, administration, diagnostic yield, patient satisfaction

## Abstract

**Background and Aims**: Colon capsule endoscopy (CCE) provides a non-invasive alternative to traditional colonoscopy. This study evaluated the feasibility, safety, diagnostic yield (DY), and patient satisfaction of the OMOM CC100 CCE system, with special focus on fully remote (n = 30) and partially remote (n = 89) administration across four centres to advance decentralised models. **Methods:** This prospective, investigator-initiated, international multicentre feasibility study enrolled 119 patients aged 18–75 years at centres in Denmark, Sweden, Portugal, and Poland from July 2024 to May 2025. Indications included rectal bleeding, iron-deficiency anaemia, a positive faecal immunochemical test, changes in bowel habit, suspected inflammatory bowel disease (IBD), post-polypectomy or colorectal cancer (CRC) surgery surveillance, and a family history of CRC. The OMOM CC100 capsule was employed with a standardised bowel preparation regimen. Administration was fully remote in Denmark using the IntelliGI™ platform and partially remote (clinic ingestion, home completion) at the other sites. Primary outcomes encompassed procedure feasibility, completion rate (capsule excretion or anal verge visualisation), bowel cleanliness (Leighton-Rex scale ≥ 3), diagnostic yield, and patient satisfaction. Secondary measures included transit times, adverse events, and technical failures. **Results**: Median age was 55.7 years (65 males, 54 females). Overall completion rate was 79%, varying by centre: Sweden (90%), Portugal (81%), Denmark (80%), and Poland (63%). Adequate bowel cleanliness was achieved in 71% of cases. Diagnostic findings included polyps (25 patients), angioectasia (20), diverticulosis (17), and mucosal inflammation (17); 42% were normal. Fully remote administration yielded 80% completion and 89.7% satisfaction. No serious adverse events occurred; overall satisfaction was 81%, with 87% preferring home-based procedures. **Conclusions:** The OMOM CC100 CCE system is feasible, safe, with DY comparable to established systems. IntelliGI™-enabled remote administration promotes decentralised care, enhancing accessibility.

## 1. Introduction

Colonoscopy is the established gold standard for evaluating colonic pathology, including polyps, inflammatory bowel disease (IBD), and colorectal cancer (CRC). Despite its diagnostic and therapeutic utility, uptake remains limited by barriers such as patient discomfort, the need for sedation, and procedural invasiveness [1]. These limitations contribute to the fact that a substantial proportion of CRC diagnoses still occur outside of structured screening programmes, often in symptomatic individuals or as emergency presentations [2]. In recent years, colon capsule endoscopy (CCE) has emerged as a less invasive alternative, offering full visualisation of the colon without the need for sedation or endoscopic instrumentation [3]. Compared with traditional colonoscopy, CCE has demonstrated a more favourable patient experience [4], a lower complication profile [5], and potential for broader accessibility, particularly in primary care or remote settings [6].

Technological improvements in capsule imaging [7], bowel preparation protocols [8], and data transmission have further enhanced the feasibility of CCE. The OMOM CC100 capsule, developed by Jinshan Science & Technology (Group) Co., Ltd., Chongqing, China, is a CE-marked diagnostic tool designed to capture high-resolution images of the entire colon [9]. The device can be deployed with a remote administration platform [10], thereby reducing dependency on in-hospital infrastructure. This study aimed to assess the feasibility, safety, diagnostic performance, and patient satisfaction associated with the OMOM CC100 colon capsule system across several centres. A particular focus was placed on evaluating the effectiveness of remote delivery models, including complete at-home administration, to determine their potential for broader implementation in routine clinical practice.

## 2. Methods

### 2.1. Study Design and Setting

This was a prospective, investigator-initiated, international multicentre feasibility study conducted at four referral centres from Denmark (Odense University Hospital, Odense), Sweden (Skåne University Hospital, Malmö), Portugal (CUF Hospitals of Oporto), and Poland (Pomeranian Medical University, Szczecin). The study protocol was standardised across sites, with minor variations permitted following local clinical practices, particularly concerning bowel preparation. An external contractor, Corporate Health International ApS (CHI ApS, Odense, Denmark), carried out the CCE in Denmark; the process involved patient communication, a video prereading by nurses, and a final reading by certified gastroenterologists. For the other 3 sites, existing local infrastructure and mechanisms were used for capsule delivery, although the reading remained centralised.

### 2.2. Participants

A total of 119 patients were enrolled between July 2024 and May 2025. Patients were consecutively recruited from routine gastroenterology referrals at each centre. Eligible patients were adults aged 18–75 years who had been referred for colonic investigation. Indications included rectal bleeding, iron-deficiency anaemia (IDA), a positive faecal immunochemical test (FIT), changes in bowel habit, suspected or known IBD without biopsy need, post-polypectomy or CRC surgery surveillance, or a positive family history of CRC.

Key exclusion criteria included the following:

- Inability to provide informed consent and understand and comply with the instructions of the remote procedure
- Extensive colonic resection (>segmental) or surgery-risking stricture
- Known or suspected bowel obstruction or strictures
- GI motility disorders or significant constipation
- Pregnancy or breastfeeding
- Known severe renal insufficiency
- MRI planned within 14 days post-ingestion
- Allergy or contraindication to bowel-prep agents

### 2.3. Colon Capsule Endoscopy

The OMOM CC100 capsule (Jinshan Science & Technology (Group) Co., Ltd., Chongqing, China) was used for all procedures. The capsule measures 31.5 × 11.6 mm weighs approximately 3 g and is equipped with two cameras and real-time imaging capability. It has a 12 h battery life and a variable frame rate of up to 35 frames per second; the frame rate adapts dynamically based on motion detection, conserving battery during static periods [11]. The associated system includes a data recorder, a wearable antenna belt, and proprietary VueSmart software for image review [11]. The package also contains a leaflet with step-by-step instructions and a video demonstration of the data recorder setup.

### 2.4. Bowel Preparation

Participants underwent a structured bowel preparation regimen based on a low-residue diet and split-dose polyethylene glycol (PEG) with ascorbic acid (either very low volume or low volume solution) [12]. The preparation protocol was explained through a standardised written leaflet with timing tables (Table 1), through a video demonstration (for the remote procedure) and a verbal explanation by trained staff during the telephone confirmation the day before the procedure. [13]. Boosters (e.g., CitraFleet) and prokinetics (prucalopride) were mandatory if real-time monitoring showed delays (gastric > 1 h or small bowel >3 h), administered on demand via nurse guidance—local variations were permitted in pre-procedure use of macrogol (Movicol) and simethicone, based on standard practice.

### 2.5. Administration Pathways

**Figure 1:**
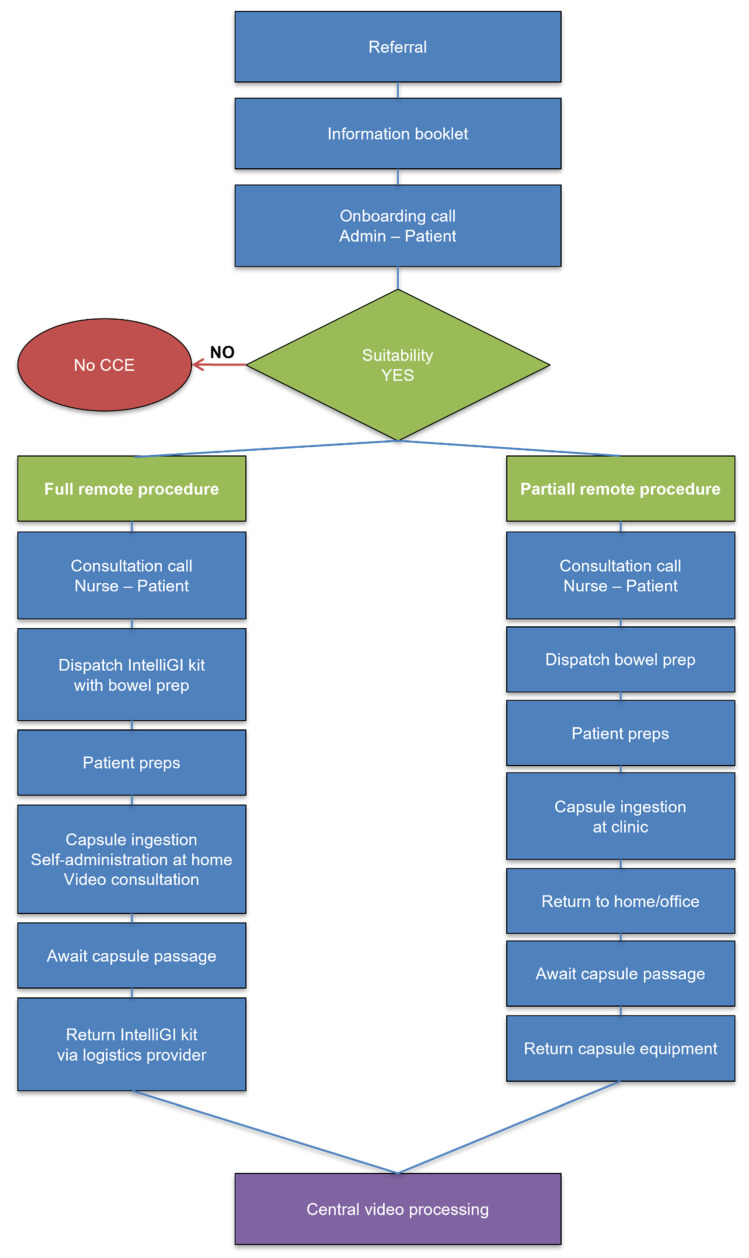
Flow chart.

In Denmark, all patients underwent complete remote administration using the IntelliGI™ kit (CHI ApS, Denmark) [6]. This included a pre-configured tablet, mobile router, secure video link with a study nurse, and GPS tracking for equipment. A 24 h helpline for troubleshooting was also available. Elderly patients were offered an optional in-person demonstration or support from a caregiver. In Sweden, Portugal, and Poland, a partially remote model was employed. Patients ingested the capsule under supervision in the clinic and completed the remainder of the procedure at home, with optional telephone support and return of equipment the next day Figure 1. This is the first multicentre OMOM CC100 trial with a full remote, partially remote, and hybrid model that extends prior single-centre reports [10], standardising across Europe.

### 2.6. Outcome Measures

The primary outcome measures of this study are summarised below:

1. Procedure feasibility: Defined as capsule ingestion and data capture without major protocol deviation.
2. Completion rate: The percentage of complete procedures (defined as capsule excreted or visualisation of the anal verge is identified without technical interruptions in the colon) reported to the total number of procedures.
3. Bowel cleanliness: Evaluated using the Leighton-Rex scale (grades 1–4) and defined adequate for scores 3–4 [14], assessed globally by a single experienced reader per centre, with 10% of cases double-read for consistency.
4. Diagnostic yield: Identification of relevant pathology (e.g., polyps, inflammatory changes, malignancy) in the large and small bowel.
5. Patient satisfaction: Assessed post-procedure using a custom 12-item questionnaire (Supplementary File S1) on Likert scales (1–5) covering overall satisfaction, ease, and preferences, adapted from prior CCE tools [15].

The secondary outcome measures of this study were GI transit times (gastric, small bowel, colonic); adverse events or complications; and equipment malfunction or technical failure.

### 2.7. CCE Video Review

All capsule videos were uploaded to a secure central server. Blinded readings were conducted by expert readers, each with experience in more than 500 CCE procedures [16]. Second reviewer (independent of initial) consulted for uncertainties (e.g., lesion ambiguity; 18% cases, n = 21/119). Only anonymised data were used during the reading and analysis phases.

### 2.8. Statistical Considerations

A total sample of 120 patients was deemed sufficient based on estimated capsule completion rates of 90%, with a 90% confidence interval and a 4.5% error margin. Descriptive statistics were used to summarise baseline characteristics, indications, cleanliness scores, and completion rates. Satisfaction responses were tabulated and reported as proportions. Although no a priori power calculation was performed for hypothesis testing due to the feasibility design, a post hoc power analysis was conducted for exploratory comparisons of completion rates across centres. Based on the observed chi-square statistic (χ^2^ = 6.49, df = 3, *p* = 0.09) and effect size (Cohen’s w = 0.23), the study had 55% power to detect this difference at α = 0.05, calculated using the non-central chi-square distribution. This limited power reflects the pilot nature of the study and informs larger future trials.

### 2.9. Ethics and Data Handling

All participants provided informed consent before inclusion. The study received ethical approval from relevant national and institutional review boards. The study complied with the Declaration of Helsinki and relevant European Union (EU) General Data Protection Regulations (GDPR). All identifiable information was excluded from central analysis. Data was stored on certified, encrypted systems (DIN 13,548 quality management standard).

## 3. Results

### 3.1. Study Population

**Figure 2:**
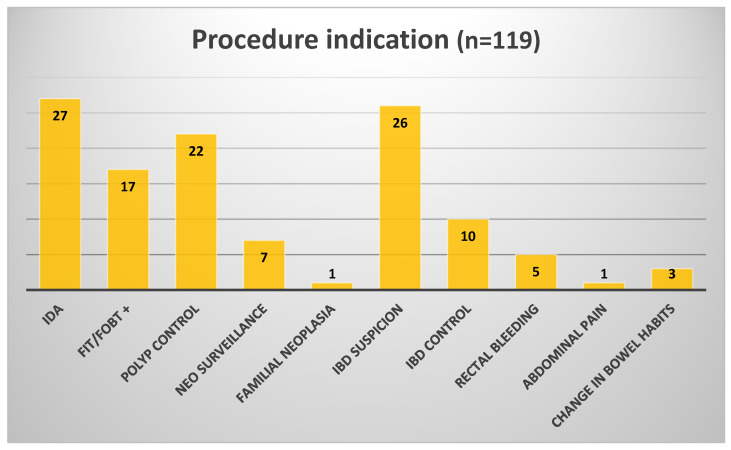
Indications for CCE (IDA—iron-deficiency anaemia; FIT—faecal immunochemical test; FOBT—faecal occult blood test; NEO—neoplasia; IBD—inflammatory bowel disease).

Of 155 approached, 36 declined (23% refusal rate) due to bowel prep related to the long-distance travel concerns (n = 20), fear of incompleteness requiring colonoscopy (n = 12), or preference for standard procedures (n = 4). This may bias toward motivated patients, limiting generalisability to broader populations. A total of 119 patients were included in the final analysis across four referral centres: Denmark (*n* = 30), Sweden (n = 30), Portugal (n = 32), and Poland (*n* = 27). The overall median age was 55.7 years, with a slight male predominance (65M/54F), Table 2. Clinical indications included suspected IBD, rectal bleeding, IDA, post-polypectomy and CRC surgery follow-up, and positive FIT, Figure 2.

### 3.2. Completion Rate

The overall completion rate was 79%. Patients with incomplete capsule studies were referred for colonoscopy or sigmoidoscopy depending on the level where the recording stopped and the bowel prep score and aligned with the clinical and local practice. The main reasons for incomplete studies were slow colonic transit and premature battery depletion. Complete CCE was achieved in 27 of 30 patients in Sweden (90%), 26 of 32 in Portugal (81.3%), 24 of 30 in Denmark (80%), and 17 of 27 in Poland (63%). Although all centres used the same core protocol, with minor local adaptations, the lower completion rate in Poland likely reflects early-phase implementation rather than protocol failure (first 15 cases: 53% vs. later cases: 75%) and a higher proportion of FIT-positive patients with motility issues (disproportionately represented among those who experienced prolonged colonic transit times). Site experience also varied, with Poland being a novice centre, which may have contributed to inter-site variability. Among the Danish incomplete CCEs (n = 6), median battery duration was 9.2 h (range 6–12 h), significantly shorter than in completed procedures (median 14 h; *p* < 0.05, Mann–Whitney test), suggesting technical factors associated with the remote setting. Overall, the 79% completion rate is consistent with the published literature. Notably, patient satisfaction was similar between incomplete and complete CCEs (mean 4.2/5 vs. 4.4/5, *p* = 0.3).

### 3.3. Bowel Cleanliness

Bowel cleanliness was assessed using the Leighton-Rex scale, with scores of 3 or 4 defined as clinically adequate. Overall, adequate bowel cleanliness was achieved in 71% of cases. The highest adequacy (80%) was observed both in Sweden and Poland, followed by Portugal with 75%, while Denmark achieved adequate preparation in 15/27 cases evaluated (55.6%). Violations (e.g., incomplete intake) occurred in 15% overall (n = 18/119), higher in remote (23%, n = 7/30) vs. partially remote (12%, n = 11/89; *p* = 0.1, chi-square), correlating with lower cleanliness.

### 3.4. Diagnostic Findings

**Figure 3:**
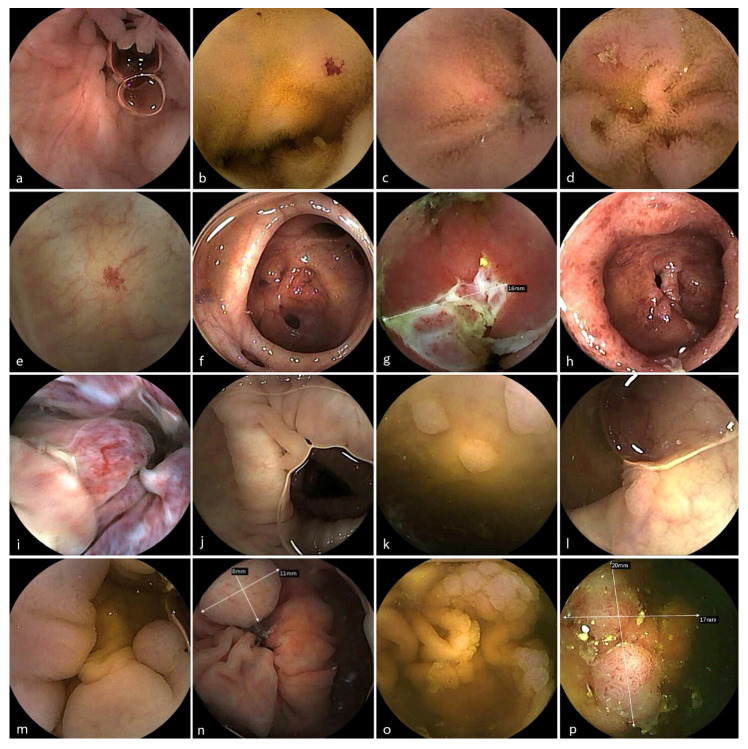
Capsule endoscopic findings in the small bowel (upper row) and large bowel. (**a**) Lymphoid hyperplasia. (**b**) Angioectasia. (**c**) Aphthoid erosion. (**d**) Crohn ulceration. (**e**) Angioectasia. (**f**) Diverticulosis. (**g**) Ulcerated Crohn stricture. (**h**) Ulcerative colitis. (**i**) Haemorrhoids. (**j**) Inflammatory pseudopolyps. (**k**) Diminutive sessile hyperplastic polyps in the rectum. (**l**) 8 mm sessile polyp. (**m**) 10 mm pedunculated polyp. (**n**) 11 mm sessile serrated polyp. (**o**) Large laterally spreading polyp. (**p**) 20 mm sessile adenomatous polyp.

CCE identified a broad range of clinically relevant findings within the small and the large bowel with an overall diagnostic yield (DY) of 58%, Table 3 and Figure 3. The DY for the remote group was 74% but with a heterogeneous distribution of the referral indications between the groups. These included

**Angioectasia:** In the data from the POR centre were described 11 patients with this type of lesion in the small bowel and 5 patients with the localisation in the colon; from the DEN centre there were also 4 patients where these vascular lesions were present in the small bowel.

**Polyps**: Commonly detected in patients undergoing surveillance, particularly in Denmark (in 19 out of 26 capsules reaching different levels of the colon—we only excluded 3 patients where the capsule did not reach the colon and one with bowel prep classified 0 on Leighton-Rex scale) and in Sweden (6/30 patients).

**Diverticulosis**: Was more commonly described in the group from Portugal—10 patients—followed by Denmark with 4 patients, and Sweden—3 patients.

**Mucosal inflammation**: Identified in patients referred for suspected or known IBD, often correlating with clinical suspicion (mainly in Portugal—14 patients had small bowel lesions, while in the Swedish cohort there were only 3 patients with observed ulcerations and erosions in the small bowel).

**Normal studies**: A significant proportion of cases (42% of patients, reaching different levels of the colon), particularly among those referred for mild symptoms or borderline indications, yielded no abnormal findings, consistent with expected diagnostic yield patterns in non-screening populations.

### 3.5. Full Remote Administration via IntelliGI™

**Figure 4:**
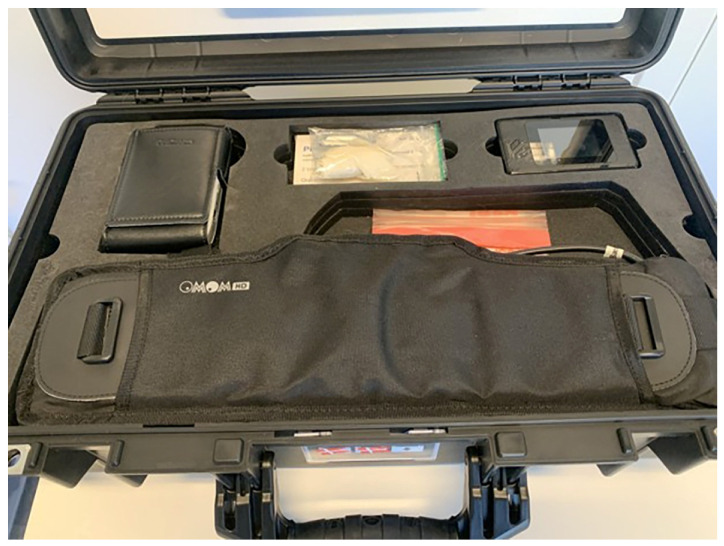
IntelliGI™ kit (CHI ApS, Denmark) Recorder and OMOM capsule, Bowel prep, Device for internet connection, Belt to wear recorder, Written information material, iPad.

The IntelliGI™ system supports complete remote administration of CCE. In Denmark, 38 eligible patients were approached for fully remote; 8 declined (21%) due to anxiety about performing the procedure independently, (n = 5) or concern about activating or managing the recorder at home (n = 3), biassing toward tech-comfortable individuals. All participants (n = 30) received the complete remote care model, including delivery of a pre-configured kit with capsule, booster agents, a tablet with secure telehealth interface, a 5G router, and a GPS tracker, Figure 4.

Among these 30 patients,

- All have successfully received and returned their IntelliGI™ kits.
- 100% completed the procedure without needing in-person assistance.
- 80% achieved full capsule completion.
- 55.6% achieved adequate cleanliness.
- No technical malfunctions or connectivity failures were reported.

Partially remote use of IntelliGI™ at all the other sites also demonstrated high reliability, allowing patients to complete procedures at home following capsule ingestion at the clinic. All 89 patients successfully returned the equipment without any reported problems. In total, 78.7% of them had complete CCE videos, and for 87 assessed CCE videos the bowel cleanliness was adequate in 78.2%, Table 4.

### 3.6. Safety and Technical Performance

No serious adverse events were reported. A small number of patients experienced mild GI symptoms, such as transient bloating or nausea related to the bowel preparation. No capsule retention occurred. All recorded video data were successfully uploaded to the secure cloud platform for central analysis. No cases of hardware or software failure were identified. There were no failures in data retrieval, connectivity, or equipment handling. These findings confirm that the IntelliGI™ platform, after appropriate education, is a robust tool for remote CCE delivery, offering high procedural success and a positive user experience.

### 3.7. Patient Satisfaction

**Figure 5:**
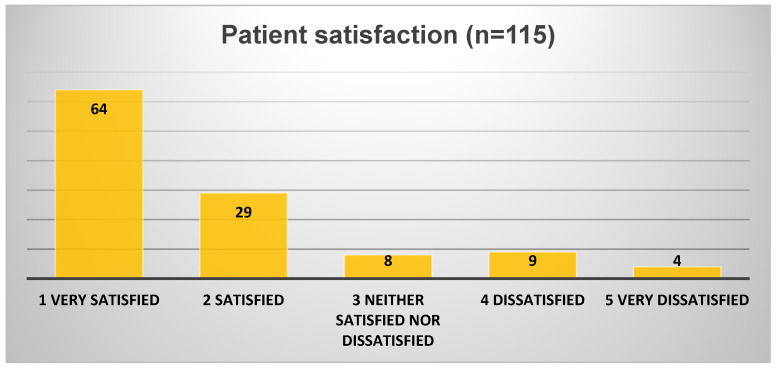
Patient satisfaction with the CCE procedure.

Patients reported high levels of satisfaction with the remote process (93 out of 115 responders; 80.9%—Figure 5) and even higher for the subgroup conducting the procedure fully remotely (26 of 29 responders—89.6%):

- 76.5% (88 of 115 responders) or 89.6% for the full remote centre (26 of 29 responders) found the setup instructions extremely clear or very clear.
- 91% found the procedure easy or very easy to complete at home.
- 87% expressed a preference for future CCE to be fully remote, home-based rather than in-hospital.

Patient feedback was uniformly positive across the centres reporting satisfaction data. Most patients found capsule ingestion manageable (only two described difficulties), and the bowel preparation tolerable (nine were dissatisfied). Among those who experienced full remote care, the majority preferred it over conventional in-hospital investigations. The procedure was described as minimally disruptive to daily activities (only two had discomfort managing equipment), and most patients stated they would agree to undergo CCE again if needed.

## 4. Discussion

This international multicentre feasibility study demonstrates that CCE using the OMOM CC100 system is a viable, safe, and well-tolerated alternative to traditional colonoscopy for a range of clinical indications. Notably, the panenteric nature of the OMOM CC100, enabling evaluation of both the small and large bowel, offers an expanded diagnostic reach that may be particularly valuable in patients with overlapping upper and lower gastrointestinal symptoms [17,18]. Furthermore, the integration of remote administration through the IntelliGI™ platform appears both technically feasible and highly acceptable to patients, marking a meaningful step toward decentralised diagnostics in gastroenterology.

Across the four participating centres, the overall completion rate and bowel cleanliness levels achieved with OMOM CCE were comparable to, and in some settings exceeded, published benchmarks for earlier-generation capsule systems [9]. Lower numbers from the Polish cohort are likely reflecting early-stage implementation rather than protocol failure. These rates support the procedural feasibility of both fully and partially remote CCE pathways using the OMOM CC100 system.

The highest performance was observed in Sweden and Portugal, where both completion and adequate cleanliness rates exceeded 65–70%. While Denmark’s bowel preparation adequacy was lower (55.6%), this was likely influenced by the greater complexity of managing a fully remote protocol and suggests that further patient support or regimen refinement may optimise outcomes. Importantly, this study confirms that CCE can be successfully administered in a fully remote, home-based model without compromising data quality, safety, or patient satisfaction. This has significant implications for healthcare delivery models, particularly in regions with limited endoscopy access, overstretched services, or where patient mobility is restricted.

The IntelliGI™ platform performed reliably in both full and partial remote models. Its successful deployment in Denmark demonstrates that patients can self-administer complex bowel preparation and capsule ingestion protocols when supported by a structured digital interface and nurse-led teleconsultation. The absence of technical failures, strong patient satisfaction, and high rates of protocol compliance support further integration of this model into real-world practice. This approach could ease pressure on hospital resources, reduce waiting times, and improve access in rural or underserved areas. The broader use of the IntelliGI™ kit also offers a framework for decentralised diagnostics in other endoscopy-adjacent domains, such as small bowel imaging, motility studies, or non-invasive screening initiatives.

Remote administration has also the potential to generate meaningful operational and economic advantages by reducing patient travel, minimising on-site staff time, and decreasing reliance on hospital facilities. These factors may translate into lower overall service costs and improved workflow efficiency within endoscopy units. However, the magnitude of these savings is partly dependent on adequate patient preparation, as suboptimal cleansing or incomplete studies may necessitate repeat procedures and thereby offset the expected cost benefits. In addition to potential financial advantages, remote pathways may also confer positive environmental effects by reducing patient transportation and on-site resource consumption. Nevertheless, these observations remain speculative, and formal cost-effectiveness and environmental impact analyses are required to quantify the true benefits of remote CCE implementation.

The diagnostic yield of CCE in this study was clinically relevant, particularly in the contexts of polyp surveillance and IBD assessment. As expected, a proportion of patients (especially those referred for vague or low-risk symptoms) had normal studies, and no small or large bowel malignancy was detected, reaffirming CCE’s role as a reliable filter test in stratified care pathways. The high tolerability and non-invasiveness of the test support its potential use in initial triage before proceeding to therapeutic colonoscopy.

Several limitations warrant consideration. First, some variability existed in indications, bowel preparation regimens, and procedural execution across sites; however, this reflects real-world practice and enhances external validity. Second, as a feasibility study, it was powered to evaluate safety and usability rather than to enable a head-to-head comparison of diagnostic performance with colonoscopy. Additional limitations include heterogeneity in administration models, with fully remote implementation confined to a single centre (Denmark) and comprising only 25% of the total cohort (n = 30), which may limit generalisability. Although the feasibility of CCE is well established, this study advances the field by demonstrating the scalability of remote models across diverse healthcare settings, addressing critical gaps in patient-centred, decentralised care. A further limitation is the absence of data on participants’ educational level. While instructions were simplified to enhance usability, the generalisability of these results across populations with varying educational backgrounds remains uncertain. Future research should address this by including measures of health literacy and education. The overall completion rate of 79% (versus the assumed 90%) widened the 90% confidence interval margin of error (6.1% vs. 4.5%), thereby reducing the precision of subgroup estimates and underscoring the need for adjusted sample sizes in future definitive studies.

The findings support further scale-up of remote CCE pathways in secondary and primary care. Larger, randomised studies comparing remote versus standard CCE administration, as well as comparisons with colonoscopy, are warranted. Additionally, refinement of patient education materials, booster regimens, and digital engagement tools may further improve cleanliness rates and reduce partial exams. In parallel, health economic evaluations should be conducted to determine the cost-effectiveness and sustainability of wide-scale implementation. Initial data suggest that remote CCE could reduce the need for hospital-based investigations in low-risk populations, offering both clinical and economic advantages.

## 5. Conclusions

This multicentre feasibility study demonstrates that colon capsule endoscopy using the OMOM CC100 system is a practical and patient-friendly alternative to conventional colonoscopy. The procedure showed high completion rates, clinically acceptable cleanliness, and reliable diagnostic performance across diverse clinical settings. The integration of a remote administration platform further underscores the potential for decentralised, home-based diagnostic pathways in gastroenterology. Patients found the process accessible, minimally disruptive, and highly acceptable, key factors for broader adoption.

## Figures and Tables

**Table 1:** Bowel preparation regimen.

Time	Preparation Regimen
**1 day before CCE**	Light breakfast followed by clear liquids
**From 5 to 7 p.m.**	0.5 L PEG + Asc (Plenvu™) + 1 L water OR 1 L PEG + Asc (Moviprep™) + 1 L water
**2–4 h before CCE**	0.5 L PEG + Asc (Plenvu™) + 1 L water OR 1 L PEG + Asc (Moviprep™) + 1 L water
**2 h before CCE**	200 mg Simethicon
**30 min before capsule ingestion**	150 mL water + 2 mg Prucalopride 200 mg Simethicone
**First hour after capsule ingestion**	Chewing gum
**1st boost:** **1 h post capsule ingestion**	Mg oxide + Na picosulfate (Picoprep™) + 1 L clear liquids
**2nd boost:** **2 h after 1st boost**	Mg oxide + Na picosulfate (Picoprep™) + 1 L clear liquids
**3rd boost:** **2 h after 2nd boost**	Coffee/tea and a small fatty snack
**4th boost:** **2 h after 3rd boost**	20 mg bisacodyl suppository

**Table 2:** Patients’ characteristics (SWE—Sweden centre; POR—Portugal centre; DEN—Denmark centre; POL—Poland centre).

	SWE	POR	DEN	POL	TOTAL
**No. patients**	30	32	30	27	119
**Median age (years)**	39.5	62.5	66	49	55.7
**Male**	11	14	22	18	65
**Female**	19	18	8	9	54

**Table 3:** Diagnostic findings.

Findings	Inflammation, Ulcer, Erosions	Angioectasia	Diverticula	Polyp	Normal Study
**Stomach**	3	1	0	0	n/a
**Small bowel**	21	15	0	0	66
**Large bowel**	9	5	18	32	52

**Table 4:** Characteristics of the complete versus partial remote procedure.

	Completion Rate	Bowel Cleanliness	Technical Issues
**Full remote CCE**	80%	55.6%	None
**Partial remote CCE**	78.7%	78.2%	None

## Data Availability

The data presented in this study are available on request from the corresponding author due to ethical reasons.

## Supplementary Materials

The following supporting information can be downloaded at: https://www.mdpi.com/article/10.3390/diagnostics16010020/s1, File S1: attached separately.
