# Supplementary material for: Prospective, Multicentre Feasibility Study of Remote Colon Capsule Endoscopy Using the OMOM CC100 System
Source: Diagnostics (Basel). 2025 Dec 20;16(1):20. doi: 10.3390/diagnostics16010020 (PMC12785793; doi:10.3390/diagnostics16010020)
Supplement: Supplementary file 1 [file diagnostics-16-00020-s001.zip › diagnostics-3991888-Supplementary Material-File S1.pdf]

Supplementary Materials

Patient satisfaction questionnaire post colon capsule endoscopy (CCE).

(please circle which of the following responses to the questions applies more to you)

1. Overall, how satisfied were you with the CCE examination?

Very satisfied

Satisfied

Neither satisfied nor dissatisfied

Dissatisfied

Very dissatisfied

2. How clear were the instructions?

Extremely clear

Very clear

Somewhat clear

Not so clear

Not at all clear

3. Using any number from 0 to 5 where 5 is extremely difficult and 0 is extremely easy, what number would you use to rate how easy it was for you to swallow the capsule?

0 1 2 3 4 5 - Extremely difficult

If yes, please describe below.

---

4. Did you require assistance from the nurse while doing the procedure?

Yes

No

If yes, why?

----

5. How likely are you to have the CCE again, if clinically indicated in future?

Very likely

Likely

Neither likely nor unlikely

Unlikely

Very unlikely

6. Did you experience any pain or discomfort during the procedure from 0 to 5? Where 5 is extremely painful and 0 is no discomfort

0 1 2 3 4 5 - Extremely difficult

7. How easy was the use of bowel prep?

Very easy – Easy - Neither easy nor difficult – Difficult - Very difficult

8. Did you manage to continue with your daily activity while having the capsule endoscopy?

Yes

No

9. Would you prefer doing the CCE in the hospital or at home?

At home

In the hospital
